# Analysis of size effect and its influencing factors of brittle red sandstone with different heights

**DOI:** 10.1038/s41598-024-66789-1

**Published:** 2024-07-09

**Authors:** Feng Chen, Jinyang Du, Chun’an Tang, Yanhong Du, Yishan Pan

**Affiliations:** 1 School of Mechanics and Engineering, Liaoning Technical University, Fuxin, 123000 Liaoning China; 2 State Key Laboratory for Geomechanics and Deep Underground Engineering, Beijing, 100083 China; 3 Institute of Rock Instability and Seismicity Research, Dalian University of Technology, Dalian, 116000 Liaoning China; 4 Environmental Engineering College, Liaoning University, Shenyang, 110000 China

**Keywords:** Rock mechanics, Uniaxial compression, Size effect, End effect, Dissipated energy density, Solid Earth sciences, Natural hazards

## Abstract

We carried out uniaxial compression tests on brittle red sandstone with different heights. The test results show that the uniaxial compressive strength of rock sample increases first and then tends to be stable with the increase of the size, which is approximately stable between 75 and 81 MPa. Both elastic energy and dissipated energy increase with the increase of rock sample size. In order to further analyze the mechanism behind these phenomena, we combined advanced numerical simulation and theoretical analysis to explain these phenomena, and systematically analyzed the end face effect as one of the key factors affecting the uniaxial compression characteristics of brittle red sandstone for the first time. Small sized rock samples are very sensitive to end effect. The middle of the large sized rock samples is in a uniform compression state, and the effect of end effect is weakend. When there are rigid pads at both ends of the rock sample, there is an obvious elastic vertebral body during the loading process of the rock sample. The bearing capacity of rock samples with rigid pads is greater than that of rock samples without rigid pads, and the energy released during instantaneous failure of rock samples without rigid pads is greater than that of rock samples with rigid pads. The findings of this paper make a valuable contribution to establishing optimal study sample sizes and advancing the utilization of laboratory test mechanics parameters in engineering applications.

## Introduction

A lot of research has been carried out on the rock mechanical properties under uniaxial compression, but the deep mechanism of crack initiation and propagation in rock has not been fully understood^1^. Theoretically, the material properties should not be affected by the geometry of the rock sample and the test conditions. However, this is not the case for the key mechanical parameters of brittle materials such as uniaxial compressive strength and elastic modulus during the test. Taking the uniaxial compressive strength measurement of rock sample as an example, both the International Society for Rock Mechanics (ISRM) and the American Society for Testing and Materials (ASTM) provide optimal size recommendations for test samples^2^. However, despite adherence to these recommendations in some tests, significant size effect still manifest in the experimental results. The widespread application of indoor experimental mechanics parameters to engineering scales continues to face substantial challenges.

The size and geometric shape of rock samples are crucial influencing factors in the study of the mechanical properties of rocks^3^. Cao et al*.* investigated the strength and failure characteristics of rocks using prefabricated joints, emphasizing the significant impact of the internal joints and their angles with the horizontal direction on the compressive strength^4^. Park et al*.* studied the occurrence and propagation of closed cracks in gypsum rock sample under uniaxial compression, suggesting similarities in the cracking processes between open and closed defects^5^. Hoek et al*.* demonstrated the rationality of applying Griffith's theory to the study of compressed fracture^6^. Li et al*.* conducted a study on predicting the failure of rock materials using finite element methods, asserting the superior performance of the Hoek–Brown criterion in predicting rock fracturing under complex stress conditions^7^. Mai et al*.* identified the presence of end effect in uniaxial compression tests on rock samples, noting their significant influence on uniaxial compressive strength, stress–strain curves, and fracture morphology, especially for smaller sample lengths^8^. Wang et al*.* Failure and energy evolution characteristics of rock-backfill composite structures (RBCS) in underground mines under alternating fatigue and creep loading^9,10^. Most scholars agree that end effect do impact the failure of rocks, and this impact varies with changes in sample size^11,12^. Because of the heterogeneous nature of rocks, the post-failure fracture patterns are complex, including but not limited to shear failure at an angle to the compressed surface and tensile failure perpendicular to the compressed surface. Scholars have addressed these issues by studying rocks of different sizes, leading to the identification of the Representative Elementary Volume (REV) characterizing the mechanical properties of rocks. For a long time, researchers have associated alterations in rock sample size with fluctuations in internal defect quantities, leading to variations in mechanical parameters—a phenomenon commonly known as the size effect^13–16^. Size effect is a prominent characteristic of brittle materials like rocks, and extensive research findings on this phenomenon have been accumulated by numerous scholars^17–26^.

In recent years, an increasing number of scholars have observed that the size effect does not show a direct correlation between the variation of a specific mechanical parameter and the size of rock samples. It is a phenomenon influenced by multiple factors such as the size, shape, end roughness, elastic modulus, and experimental conditions of the rock sample^27,28^. Chen et al*.* found through the uniaxial compression experiment of rocks that the strength of rocks increased with the size of samples, and believed that other factors affected the size effect of rocks^27^. Prior experimental investigations have elucidated that, with a decrease in rock sample size, the variability in strength becomes more conspicuous. With a decrease in rock sample size, the more conspicuous the variability in strength becomes, as elucidated by prior experimental investigations. Conversely, larger rock samples exhibit reduced strength variability due to the influence of the probability distribution of internal defects. In such cases, the main factor affecting the variation of rock strength with size stems from differences in the stiffness of the testing machine's platen^29–31^. Therefore, the impact of the testing machine platen on the ends of rock sample should not be overlooked. According to the Saint–Venant principle, for small sized rock sample with a high diameter-to-length ratio, the method of applying end loads and the size of the rock sample significantly influence the stress distribution within the rock sample. Studies by Wei^32^, Wang et al.^33^, and Bieniawski^34^, utilizing laboratory tests or numerical simulations, confirm that rock samples with small length-to-width ratios are significantly affected by end effect. The aforementioned research highlights that the size effect is not merely a linear correlation between a specific mechanical parameter and changes in size.

In summary, many scholars have not comprehensively considered the impact of the testing machine platen stiffness on the ends of samples in the study of size effect on materials. For heterogeneous and brittle materials such as red sandstone and granite, the end effect generated during the uniaxial compression process have a significant influence on mechanical parameters such as stress and strain for samples of different sizes. To thoroughly investigate the size effect of brittle materials, this paper employs a comprehensive approach, including laboratory tests, numerical simulations, and theoretical analyses, to study the size effect and influencing factors of red sandstone samples with varying heights. The research analyzes how changes in sample size affect rock strength, dissipated energy density, and failure modes, while also discussing the interaction between size effect and end effect.

## Uniaxial compression laboratory test

### Rock sample preparation

**Figure 1:**
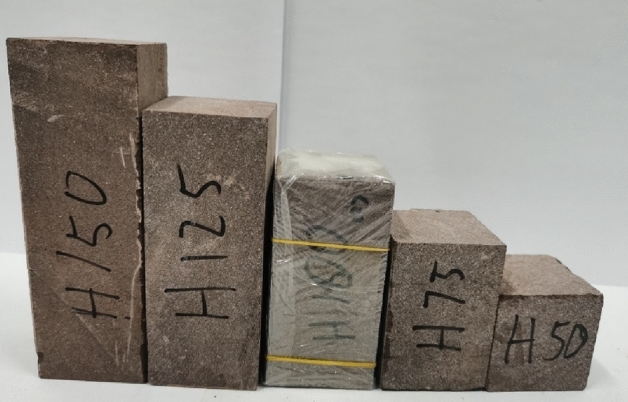
Red sandstone rock sample and its label.

**Figure 2:**
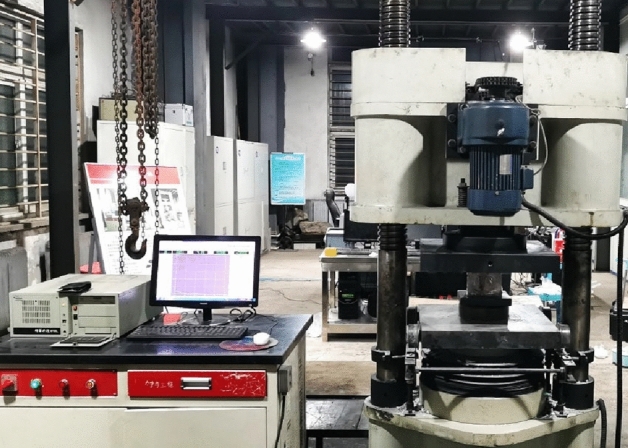
Fully automatic servo press system.

We conduct a series of uniaxial compression tests using brittle red sandstone to study the uniaxial compressive strength, rock fracture form, crack propagation and energy dissipation law of rock samples with different heights. The length and width of each rock sample are 50 mm, and the heights are 50 mm, 75 mm, 100 mm, 125 mm and 150 mm respectively (see Fig. 1). Each rock sample is marked as H50, H75, H100, H125 and H150 respectively for the convenience of description. All rock samples have no visible cracks. The average density of the rock sample is 2.425 g/cm^3^. All rock samples are wrapped with plastic film to prevent rock fragments from popping out due to sudden destruction during the test, in order to ensure integrity of the rock samples after destruction, and to ensure the test is conducted safety. The loading rate used in this test is 0.24 mm/min. In order to ensure the accuracy of the test data, three sets of tests of the same type were set for each group of samples. The fully automatic servo press system used in the test is shown in Fig. 2.

### Stress variation characteristics

**Figure 3:**
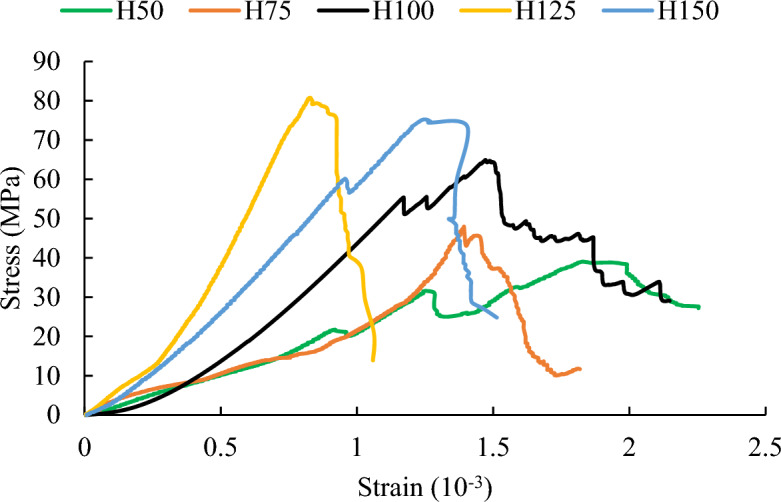
Stress–strain curves of rock samples with different heights.

**Figure 4:**
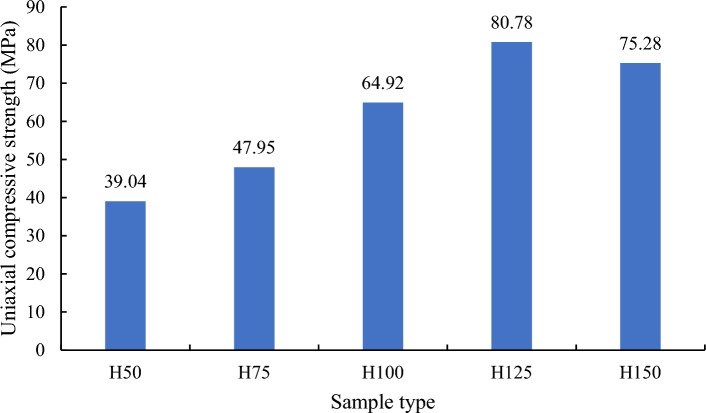
Variation trend of uniaxial compressive strength of all rock samples.

The stress–strain curve of each rock sample has experienced four stages: compaction, elastic deformation, plastic deformation and failure, as shown in Fig. 3. Some studies suggest that the number of internal defects in the rock sample will correspondingly increase and the uniaxial compressive strength should gradually decrease as the size of the rock sample increases^35–37^. However, Fig. 4 illustrates that with the increase in rock sample size, the uniaxial compressive strength gradually increases and then stabilizes (the uniaxial compressive strength of H125 and H150 roughly stabilizes between 75 MPa ~ 80 MPa). Based on the aforementioned experimental observations, we will further analyze the size effect of red sandstone and its influencing factors through numerical simulations and theoretical analysis.

### Failure form of rock sample

**Figure 5:**
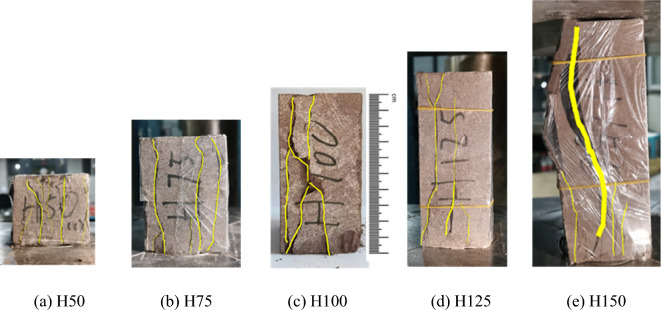
Failure form of rock samples with different heights.

**Figure 6:**
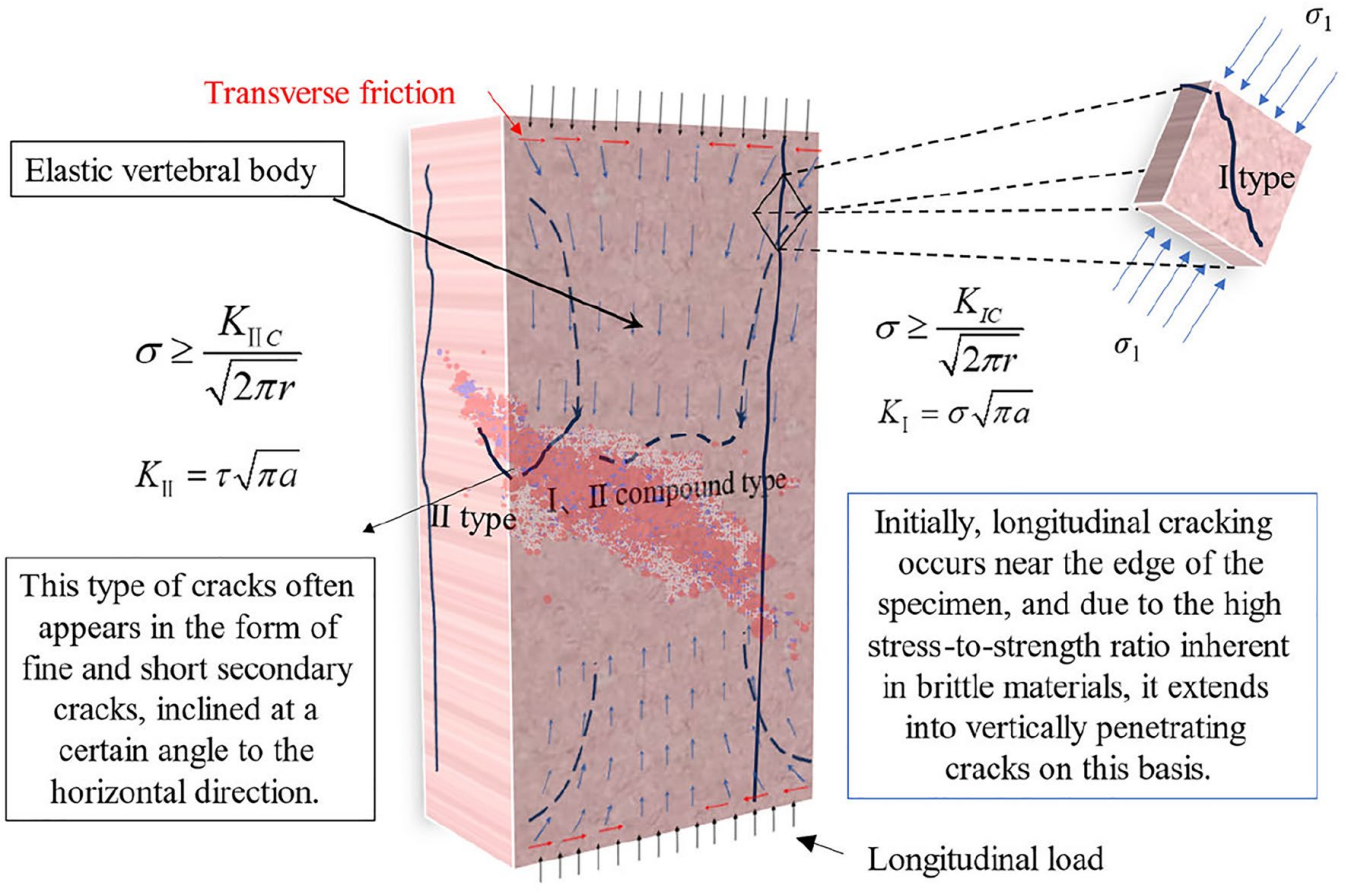
3D rock sample fracture morphology analysis and stress distribution schematic diagram. *K*_*I*_ and *K*_*II*_ b respectively represent the stress intensity factors at the tips of type I and type, respectively. *r* and *θ* denote the polar coordinates near the crack tips, *α* represents half of the crack length.

In uniaxial compression tests influenced by end effect, all rock samples exhibited vertical cracks (see Fig. 5). Due to variations in both the magnitude and direction of the maximum principal stress, crack initiation occurred through three main modes: opening-type cracks (Type I) under tensile stress orthogonal to the crack plane; sliding-type cracks (Type II) under shear stress perpendicular to the crack plane within the crack surface; and combined Type I and Type II cracks at an angle to the principal stress (see Fig. 6). For the combined Type I and Type II cracks, it is assumed that they initiate along the direction of the maximum circumferential stress in the fracture process zone, and further crack propagation occurs when the circumferential stress in this direction reaches a critical value.

### Dissipated energy change law

**Figure 7:**
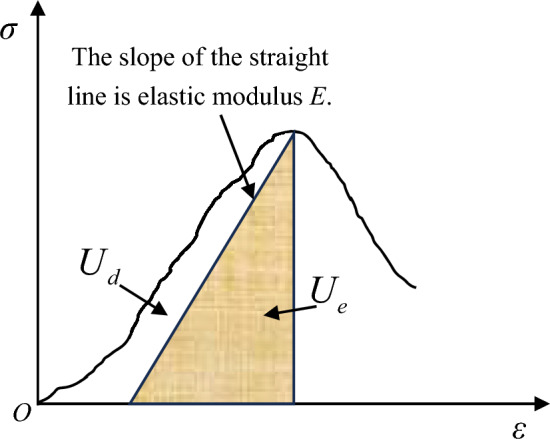
Quantitative relationship of elastic energy density and dissipated energy density.

The complexity of the internal components of rock mass determines the diversity of energy conversion during the failure process. According to the proportion of various energy before rock failure, energy can be divided into elastic energy and dissipated energy, as shown in Fig. 7.

When rocks undergo deformation under external forces, assuming no heat exchange with the external environment during the deformation process, the total input energy generated by external force work is denoted as *U*. This is in accordance with the first law of thermodynamics and the constitutive equations for rock stress and strain^38,39^.

1 $$ U = U_{d} + U_{e} $$

2 $$ \sigma = E\varepsilon e^{{( - \varepsilon /\varepsilon_{0} )}} $$

In Eq. (2):

3 $$ \begin{gathered} U = \mathop \smallint \nolimits_{0}^{{\varepsilon_{c} }} \sigma d\varepsilon = \mathop \smallint \nolimits_{0}^{{\varepsilon_{c} }} E\varepsilon e^{{ - \frac{\varepsilon }{{\varepsilon_{0} }}}} d\varepsilon \hfill \\ \hfill \\ \end{gathered} $$

4 $$ U_{e} = \frac{1}{2}\sigma_{c} \varepsilon_{e} = \frac{{\sigma_{c}^{2} }}{2E} $$

In Eq. (1) to (4), *U*_*d*_ represents dissipated energy density. *U*_*e*_ represents elastic energy density. *ε*_*e*_ represents elastic strain. *σ*_*c*_ represents uniaxial compressive strength. *ε*_*c*_ represents peak strain.

Dissipated energy is utilized for the formation of internal damage and plastic deformation within the rock mass. Its variation adheres to the second law of thermodynamics, meaning that changes in the internal state follow the trend of increasing entropy.

Expanding Eq. (3), we can obtain the absorbed energy *U* when the uniaxial compressive strength of the rock sample reaches its maximum.

5 $$ \begin{gathered} U = E\left( {\left. { - \varepsilon_{0} \varepsilon e^{{ - \frac{\varepsilon }{{\varepsilon_{0} }}}} - \varepsilon_{0}^{2} e^{{ - \frac{\varepsilon }{{\varepsilon_{0} }}}} } \right|_{0}^{{\varepsilon_{c} }} } \right) \hfill \\ = E\left[ {\varepsilon_{0}^{2} - \varepsilon_{0} \left( {\varepsilon_{c} - \varepsilon_{0} } \right)e^{{ - \frac{{\varepsilon_{c} }}{{\varepsilon_{0} }}}} } \right] \hfill \\ \end{gathered} $$

Substituting the constitutive relationship into Eq. (2) yields the equation for elastic energy density:

6 $$ \begin{gathered} U_{e} = \frac{1}{2}\sigma_{c} \varepsilon_{e} \hfill \\ = \frac{1}{2}E\varepsilon_{c}^{2} e^{{ - \frac{{2\varepsilon_{c} }}{{\varepsilon_{0} }}}} \hfill \\ \end{gathered} $$

The equation for dissipated energy density is as follows:

7 $$ \begin{gathered} U_{d} = U - U_{e} \hfill \\ = ( - E\varepsilon_{0} \varepsilon_{c} - E\varepsilon_{0}^{2} )e^{{ - \frac{{\varepsilon_{c} }}{{\varepsilon_{0} }}}} - \frac{1}{2}E\varepsilon_{c}^{2} e^{{ - \frac{{2\varepsilon_{c} }}{{\varepsilon_{0} }}}} + E\varepsilon_{0}^{2} \hfill \\ = [ - E\varepsilon_{0} (\frac{{\sigma_{c} }}{E} + \frac{{\sigma_{c} }}{YDx}) - E\varepsilon_{0}^{2} ]e^{{ - (\frac{{\sigma_{c} }}{{E\varepsilon_{0} }} + \frac{{\sigma_{c} }}{{YDx\varepsilon_{0} }})}} - \frac{1}{2}E(\frac{{\sigma_{c} }}{E} + \frac{{\sigma_{c} }}{YDx})^{2} e^{{ - 2(\frac{{\sigma_{c} }}{{E\varepsilon_{0} }} + \frac{{\sigma_{c} }}{{YDx\varepsilon_{0} }})}} + E\varepsilon_{0}^{2} \hfill \\ \end{gathered} $$

Using Eqs. (5), (6), and (7), the corresponding energies can be calculated as shown in Table 1. For the small sized rock sample H50 with a relatively small length-to-width ratio, the dissipated energy accounts for a larger proportion, reaching 93.35%. In contrast, the large sized rock sample H150 with a greater length–width ratio has a dissipated energy proportion of 89.90%. Rock sample H50 exhibits more shear type cracks, and since shear type cracks need to overcome surface energy and perform work against friction during their extension, the energy consumption associated with the generation of shear type cracks is greater than that of tensile type cracks.

**Table 1:** Energy values and proportions for various-sized rock samples.

Rock sample number	Total energy density J/cm^3^	Elastic energy density J/cm^3^	Dissipated energy density J/cm^3^	Elastic energy ratio %	Dissipated energy ratio %
H50	0.19	0.18	0.01	93.35	6.65
H75	0.23	0.21	0.02	91.75	8.25
H100	0.38	0.35	0.03	92.22	7.78
H125	0.38	0.34	0.04	91.05	8.95
H150	0.45	0.4	0.05	89.90	10.10

**Figure 8:**
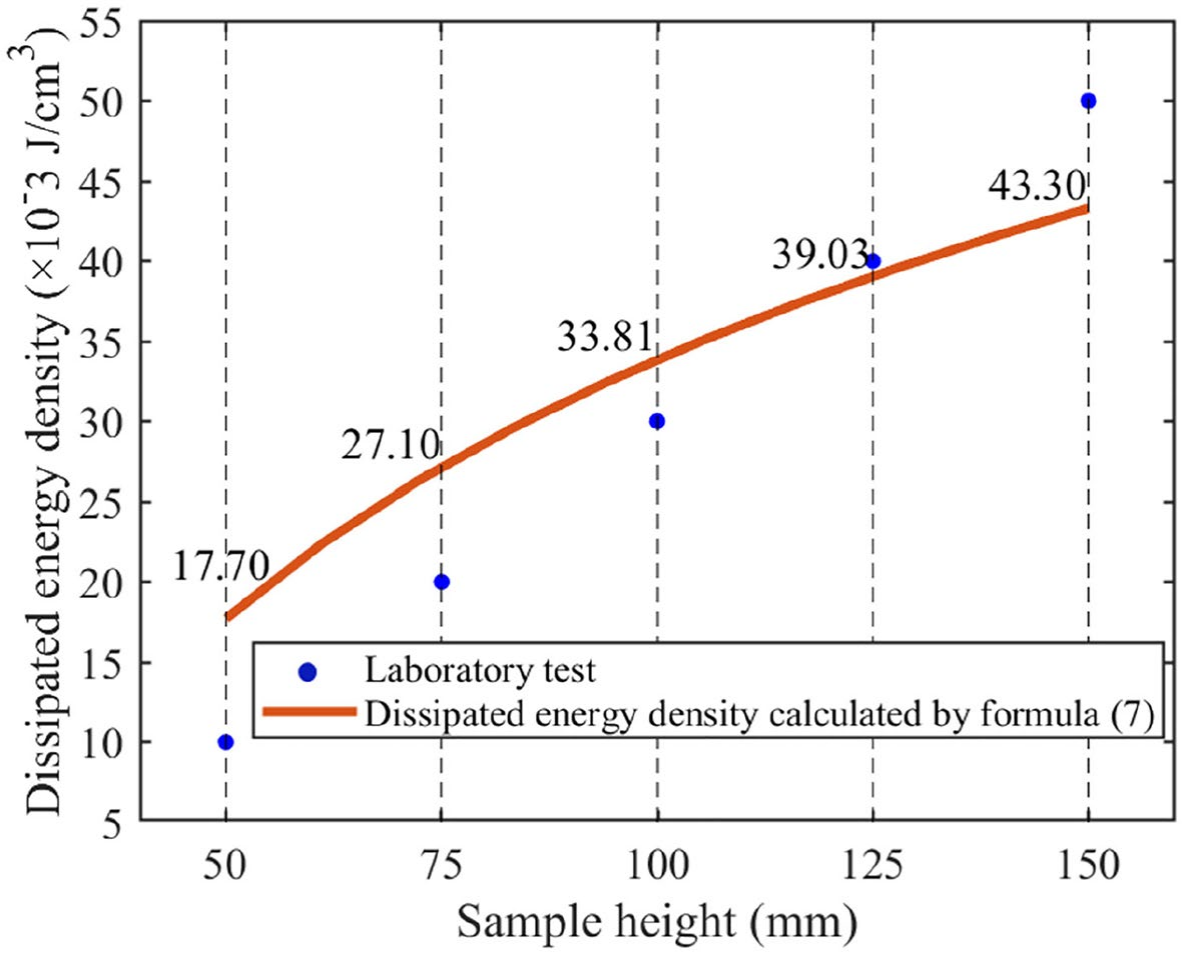
Comparison between the dissipated energy calculated using Eq. (7) and experimental data.

Through theoretical analysis and laboratory tests, it is observed that the dissipated energy during the rock failure process increases with the rock height (see Fig. 8). We conducted Pearson correlation coefficient analysis between the model and the test data, and the result was 0.987133, which indicates that there is a high linear positive correlation between the calculated results and the direct results. According to the definition and interpretation of Pearson correlation coefficient, this value is close to 1, indicating that the correlation between the two is very strong. However, the results of theoretical calculations are higher than those obtained from laboratory tests when the height is less than 125 mm. This discrepancy arises from the ideal conditions assumed in theoretical calculations, whereas the actual uniaxial compression process is influenced by end effects and material defects, leading to a reduction in dissipated energy. Particularly, the impact of end effect results in reduced energy dissipation during the tensile failure of the rock sample, significantly decreasing the energy expended in the failure process. This phenomenon diminishes when the height-to-width ratio of the rock sample is relatively large. The next chapter will provide a detailed analysis of the influence of end effect on the experimental results.

## Numerical simulation of uniaxial compression

### Brief introduction of RFPA^3D^ software

**Figure 9:**
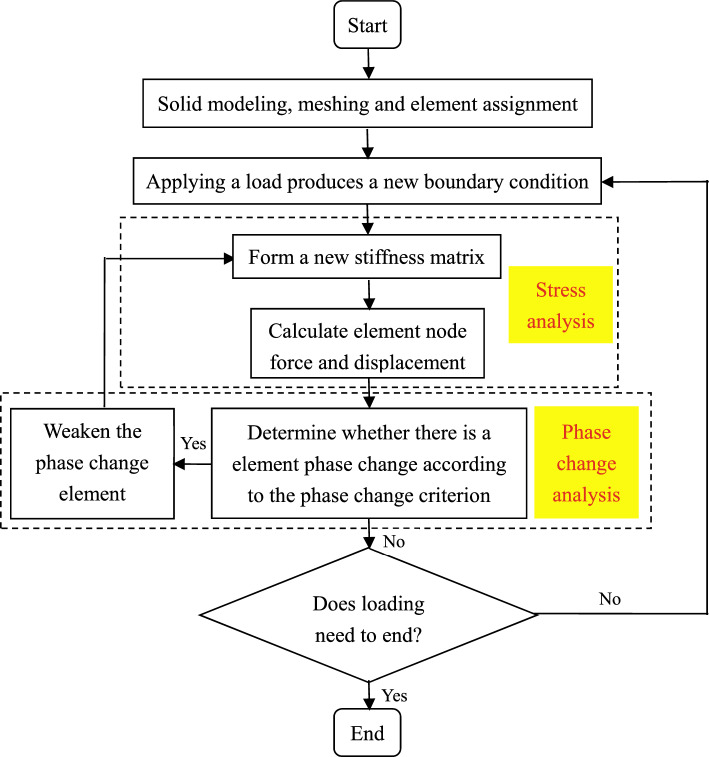
Entire workflow of RFPA^3D^ software.

We employ RFPA^3D^ (Rock Failure Process Analysis System) software to analyze the factors influencing the size effect in rock masses. RFPA^3D^ utilizes the Weibull probability distribution function to simulate rock heterogeneity and employs the Mohr–Coulomb failure criterion to determine whether rock failure occurs. The software utilizes stiffness degradation for failed elements, allowing for the application of continuum mechanics to address discontinuities in the medium. The entire workflow is illustrated in Fig. 9.

### Numerical model

**Figure 10:**
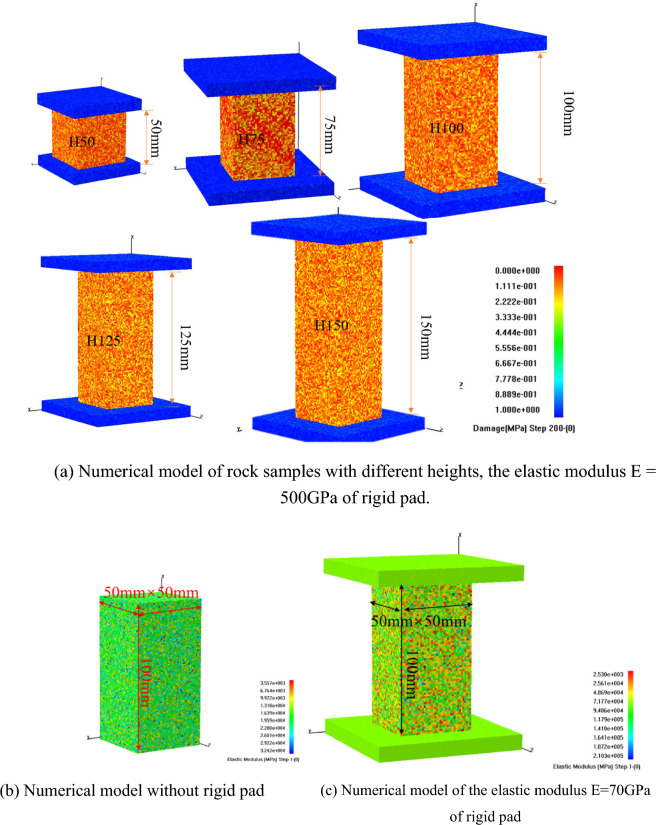
Numerical model.

For ease of comparison with uniaxial compression laboratory tests on red sandstone of different heights, a numerical model is established as shown in Fig. 10a. The model dimensions match the actual rock sample sizes, with the dimensions of the loading surface being 50 mm × 50 mm, and heights of 50 mm, 75 mm, 100 mm, 125 mm, and 150 mm, respectively. Each numerical model consists of cubic elements with a side length of 1.25 mm. To ensure consistency between numerical simulations and laboratory tests, platen boundaries are set at both ends of the model. To investigate the impact of end effect with and without platens of different stiffness, numerical models without platen, and with platens of varying stiffness are established, as illustrated in Fig. 10b and c. Different platen stiffness is achieved by altering the elastic modulus of the platens.

The RFPA^3D^ software uses the parameter *m* to reflect the irregularity of defect distribution in the material. A higher *m* value indicates a more uniform distribution of mechanical properties in the material, while a lower *m* value (homogeneity coefficient) indicates a less uniform distribution. In this model, the rock sample's elastic modulus *E* is 70 GPa, Poisson's ratio *ν* is 0.25, and *m* is set to 2. The platen has *ν* = 0.27 and *m* = 50. The simulation process adjusts the loading steps between 50 ~ 200 steps based on the model size, with each step loading an increment of 0.002 mm.

### Analysis of numerical simulation results

#### Variation characteristics of stress and acoustic emission (AE)

**Figure 11:**
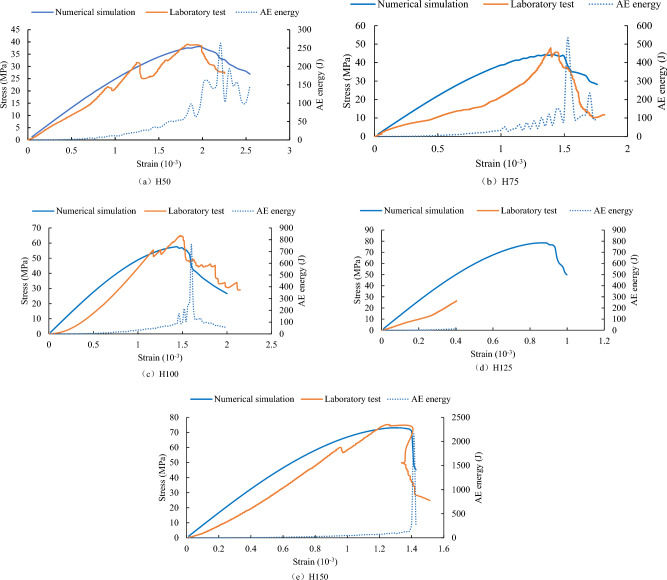
The comparison of stress–strain curves between numerical simulation and laboratory experiments, along with the AE energy curves obtained through numerical.

From Fig. 11, it is evident that the stress–strain curves obtained through numerical simulation have a good fit with the laboratory experimental results. This indicates that the RFPA^3D^ software can effectively simulate the uniaxial compression laboratory test process for rocks. Moreover, it suggests that the physical mechanical parameters obtained through numerical simulation, such as stress, strain, elastic modulus, and dissipated energy, can be used to study the size effect in rocks.

**Figure 12:**
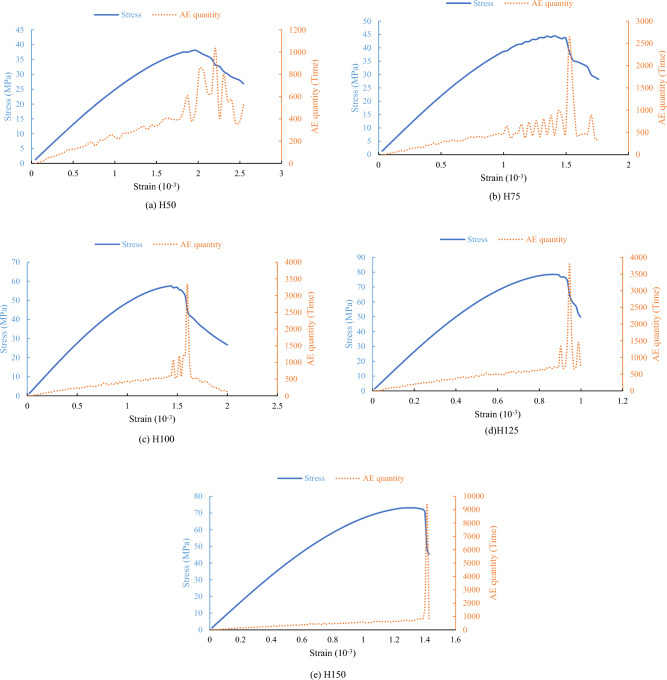
Stress and AE quantity curves obtained through numerical simulation for five types of rock samples.

Comparing laboratory experimental and numerical simulation results, it is observed that due to the inherent randomness of defects in the rock sample, the phenomenon of "stress drop" occurs suddenly during the laboratory test. However, based on the final numerical simulation results, the occurrence of "stress drop" does not significantly impact the peak stress (uniaxial compressive strength) of the rock sample and the corresponding peak strain. Before the stress reaches the peak strength, the number of AE and the AE energy do not monotonically increase but exhibit a distinct fluctuation, as shown in Figs. 11 and 12. The maximum values of AE energy often occur in the post-peak stress drop phase.

We note that the acoustic emission energy oscillates before the peak stress and reaches a maximum after the peak stress. Under stress loading, acoustic emission energy captures changes in a material's microstructure and damage accumulation. Prior to peak stress, the material undergoes elastic deformation with internal microstructure experiencing stress without notable damage. This causes oscillations in acoustic emission energy, likely stemming from uneven stress distribution, micro-defect activation, and localized stress concentrations.

Upon reaching peak stress, the material's stress distribution reaches its limit, triggering significant damage or failure in its microstructure. This releases energy in the form of acoustic emission, resulting in a sharp rise in acoustic emission energy to its peak. This indicates that peak stress marks a critical transition from elastic to plastic deformation or failure, serving as a turning point for damage accumulation and release within the material.

**Figure 13:**
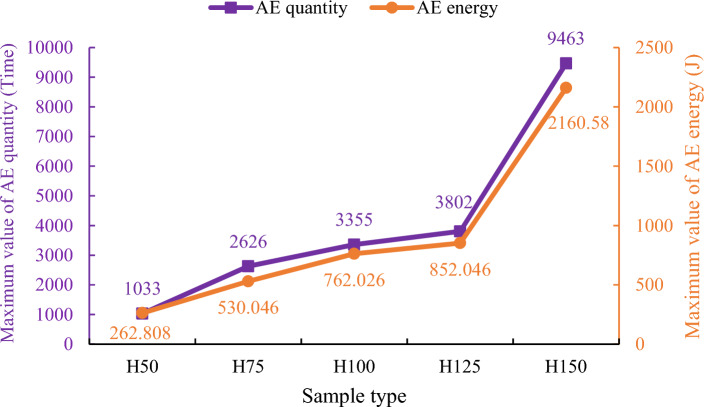
Comparison of maximum values between AE quantity and AE energy.

When the height of the rock sample is between 50 and 125 mm, the maximum values of both AE quantity and AE energy for each type of rock sample show a nearly linear increase. However, when the height of the rock sample reaches 150 mm, there is a significant increase in the maximum values of AE quantity and AE energy (see Fig. 13), indicating a more intense instantaneous failure of the rock sample. This suggests that when the rock sample size reaches a certain height, other factors, such as changes in fracture patterns, may influence the occurrence of AE events.

#### Comparison of failure characteristics of rock samples with and without rigid pads.

The end effect is analogous to the Saint–Venant principle in classical elastoplastic theory, they both focus on the distribution of stresses near the point of load application.

During the uniaxial compression process, the stress distribution in rock sample with a relatively small height-to-width ratio is influenced by the end effect. The Saint–Venant principle suggests that the influence range of end forces is approximately the size of the sample ends. So, when the height-to-width ratio exceeds 2, the stress distribution in the middle of the rock sample can be considered uniform^40^. However, factors such as the difference in stiffness and the friction of the contact surface between the testing machine platens and the rock sample in actual uniaxial compression tests can enlarge the impact range of the end effect. According to the laboratory tests mentioned in the previous section, the uniaxial compressive strength of the rock sample stabilizes when the height-to-width ratio reaches 2 to 2.5. Therefore, the end effect influences the strength, energy, and failure mode of brittle materials. We examines the impact of the end effect on brittle rock sample using the internationally standardized H100 rock sample as an example.

**Figure 14:**
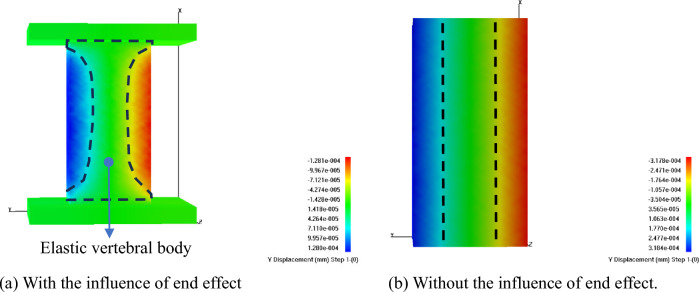
Comparison of displacement images for H100 with and without the influence of end effect.

**Figure 15:**
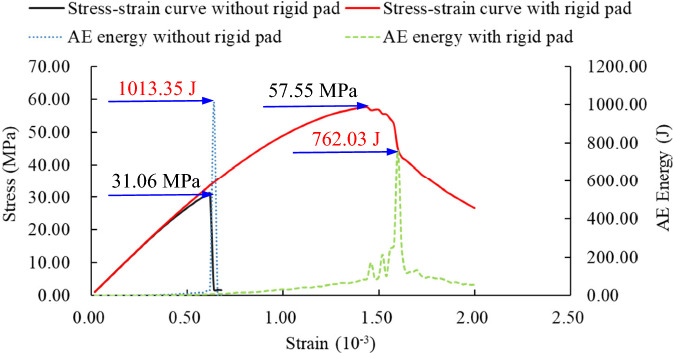
Comparison of stress, strain, and energy data for H100 with and without the influence of end effect.

The displacement cloud map of the H100 obtained through numerical simulation (see Fig. 14) reveals that when rigid pads are present at both ends of the rock sample, there is a noticeable elastic vertebral body during the loading process^26^. In contrast, when there are no rigid pads at both ends, the displacement in the rock sample exhibits a banded distribution. The uniaxial compressive strengths of the rock samples with and without rigid pads are 57.55 MPa and 31.06 MPa, respectively (see Fig. 15), indicating that the load-bearing capacity of the rock sample with rigid pads is greater than that without. The maximum AE energy values for the with and without rigid pads are 762.03 J and 1013.35 J, respectively, signifying that the rock sample without rigid pads releases more energy upon instantaneous failure compared to the one with rigid pads.

#### End effect at different stages of uniaxial compression.

**Figure 16:**
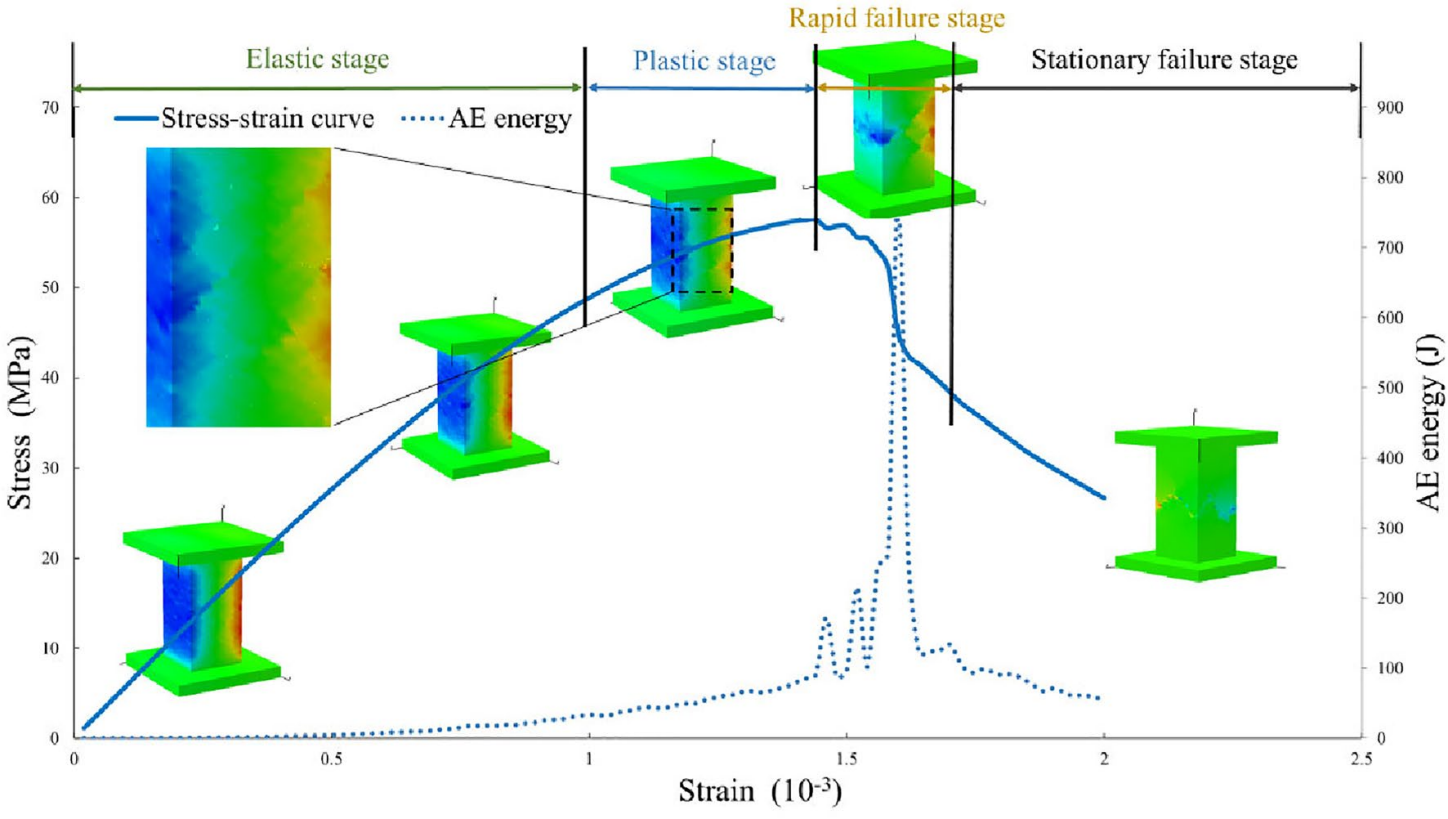
Schematic diagram of elastic vertebral body of H100 rock sample during uniaxial compression.

Based on the analysis in the previous section, it is evident that end effect during the uniaxial compression process play a significant role in the rock sample's failure mode and mechanical parameters. According to Fig. 16, the stress–strain curve of the H100 rock sample during uniaxial compression can be divided into four stages: elastic stage, plastic stage, rapid failure stage and failure stage. The failure stage can be further subdivided into rapid failure and stationary failure stages.

#### Elastic stage

In the early elastic stage, the elastic vertebral body inside the rock sample is stable with a clear boundary, and AE energy release is nearly zero. As stress increases, the shape of the elastic vertebral body remains unchanged.

In the later elastic stage, cracks start to appear in some regions, causing stress redistribution. At this point, the boundary of the elastic vertebral body inside the rock sample is relatively clear, with no significant volume change. AE energy gradually increases.

#### Plastic stage

The rock sample undergoes evident plastic deformation, cracks continuously expand, and the boundary of the elastic vertebral body becomes blurry and irregular. The overall volume of the rock sample significantly collapses. AE energy increases rapidly with intense fluctuations.

#### Rapid failure stage

The rock sample undergoes significant failure, and the main crack rapidly penetrates. The load-bearing capacity of the rock sample sharply decreases, and the elastic vertebral body essentially disappears. AE energy value suddenly rises at the point of the maximum slope in the stress–strain curve after the peak and then rapidly drops.

#### Stationary failure stage

With continued loading, the residual strength of the rock sample is slowly consumed. The failure of the rock sample becomes stable, and AE energy gradually decreases. There is no elastic vertebral body inside the rock sample.

From the above analysis, it is evident that in brittle materials, before reaching peak strength, the elastic vertebral body triggered by end effect can exhibit phenomena such as interface blurring and volume collapse. This phenomenon can serve as a crucial means for predicting rock failure.

## Interaction between end effect and size effect

### Factors influencing end effect

The discussion above indicates that end effect have a significant impact on the results of uniaxial compression tests. The following section delves into the influencing factors of end effect in uniaxial compression.

### The influence of sample size on end effect

**Figure 17:**
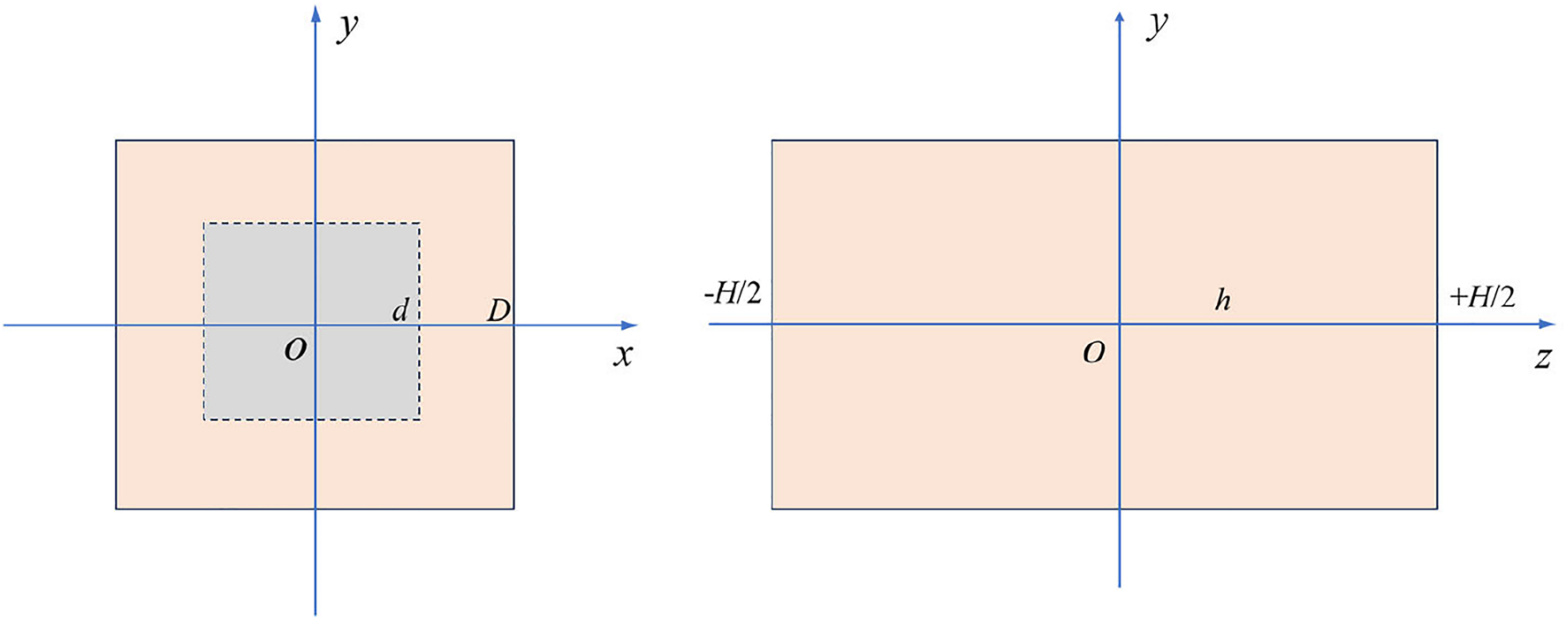
Mechanical model illustrating the influence of sample size on end effect: the orange region represents the force applied by the press to the end face of the sample, where D represents the width of the sample. For ease of calculation, the area of the sample's reaction force to the press is simplified to a gray square with a side length of d.

Firstly, establish a mechanical model illustrating the influence of sample size on end effect, as depicted in Fig. 17.

On the lateral surface x = D of the sample,

8 $$ \sigma = 0,\,\,\,\tau_{xy} = 0,\,\,\,\tau_{xz} = 0 $$

On the sample end face z =  ± H/2,

9 $$ \tau_{zx} = \tau_{zy} = 0 $$

10 $$ \sigma_{z} (x) = \left\{ \begin{gathered} \frac{F}{{D^{2} }} - \frac{F}{{d^{2} }}\begin{array}{*{20}c} {} & {} \\ \end{array} \left[ {0,d} \right] \hfill \\ \frac{F}{{D^{2} }}\begin{array}{*{20}c} {} & {} & {} \\ \end{array} (d,D] \hfill \\ \end{gathered} \right. $$

When neglecting the influence of friction on the end effects and assuming the rock sample is a homogeneous and isotropic material, the relationship between strain and displacement is as follows:

11 $$ \varepsilon = \frac{1}{2}[(\nabla u)^{{\text{T}}} + \nabla u] $$

$u = u_{x} e_{x} + u_{y} e_{y} + u_{z} e_{z}$, $\nabla u$ is displacement gradient tensor; $e = \nabla \cdot u$, is volumetric strain.

Introducing Hooke's Law:

12 $$ \sigma_{ij} + 2G\varepsilon_{ij} + \lambda \varepsilon_{kk} \delta_{ij} $$

In Eq. (12):* i、j、k* = *x, y, z* , *λ* is lame constant, $\lambda = 2\mu G/(1 - 2\mu )$, *G* is shear elastic modulus; $\delta_{ij}$ is Kronecker delta function; *σ* and *ε* respectively represent the stress tensor and strain tensor.

13 $$ \left\{ \begin{gathered} \varepsilon_{x} = \varepsilon_{y} = \frac{{\partial u_{x} }}{\partial x},\,\,\varepsilon_{z} = \frac{{\partial u_{z} }}{\partial z} \hfill \\ \varepsilon_{xy} = \varepsilon_{yx} = \frac{1}{2}\left( {\frac{{\partial u_{x} }}{\partial x} + \frac{{\partial u_{y} }}{\partial y}} \right) \hfill \\ \varepsilon_{xz} = \varepsilon_{yz} = \frac{1}{2}\left( {\frac{{\partial u_{x} }}{\partial x} + \frac{{\partial u_{z} }}{\partial z}} \right) \hfill \\ \end{gathered} \right. $$

Wei et al*.* solve this by introducing two displacement functions *Φ* and* Ψ*^29^. Due to being an axisymmetric model in rectangular coordinates, the stress components induced by displacements *u* and *v* are equal, hence Ψ = 0.

We have:

14 $$ \left\{ \begin{gathered} u_{x} = u_{y} = \frac{{\partial^{2} \Phi }}{\partial x\partial z} \hfill \\ u_{z} = - [2(1 - \mu )\nabla_{1} \Phi + (1 - 2\mu )\frac{{\partial^{2} \Phi }}{{\partial z^{2} }}] \hfill \\ \end{gathered} \right. $$

And the *Φ* is:

15 $$ \Phi = - \frac{1}{2G}\left\{ \begin{gathered} \frac{{A_{0} }}{6}z^{3} + \frac{{C_{0} }}{2}zx^{2} + \sum\limits_{m = 1}^{\infty } {\frac{1}{{\eta_{m}^{3} }}\left[ {A^{(k)} x\frac{{\partial I_{0} \left( {\eta_{m} x} \right)}}{\partial x} + B_{m}^{(k)} I_{0} \left( {\eta_{m} x} \right)]_{{\cos \left( {\eta_{m} x} \right)}}^{{\sin \left( {\eta_{m} z} \right)}} } \right]} + \hfill \\ \sum\limits_{s = 1}^{\infty } {\frac{1}{{\gamma_{s}^{3} }}} \left[ {C_{{S\;\cosh \left( {\gamma_{s} Z^{Z} } \right)}}^{{\left( K \right)\sinh \left( {\gamma_{s} Z^{Z} } \right)}} + D_{S}^{\left( K \right)} \gamma_{{S\;\cosh \left( {\gamma_{s} Z^{Z} } \right)}}^{{\sinh \left( {\gamma_{s} Z^{Z} } \right)}} } \right]J_{0} (\gamma_{s} x) \hfill \\ \end{gathered} \right\} $$

In Eq. (15): *k* = 1,2, it is determined by the boundary conditions; *J*_*0*_ and* I*_*0*_ are zeroth order Bessel function and zeroth order modified Bessl function respectively. $\eta_{m}$ = mπ/D, m is constant. $\gamma_{s}$ = *λ*/D, *λ* is the positive zero root of $J_{0} (x) = 0$. When *k* = 1, *Φ* is an odd function with respect to *z*; when *k* = 2, *Φ* is an even function with respect to* z*.

The above derivation reveals that the transverse dimensions of the sample are critical mechanical parameters affecting the stress and strain distribution near the ends. Regarding how to solve the displacement function and determine the undetermined coefficients *A*_*0*_、*B*_*m*_、*C*_*0*_、*C*_*s*_ and *D*_*s*_ based on the boundary conditions, and thereby obtain solutions for stress and strain near the ends, numerous solution methods have been provided in references^32,41–43^. We just discuss the functional relationship between the stress and strain distribution at the ends of square samples and the sample dimensions.

Through a study on the influence of end effects on cylindrical samples, reference^29^ found in the absence of other factors such as loading rate and end friction, the distribution pattern of end effects caused by variations in the transverse dimensions of the sample is consistent with the Saint–Venant principle. The strain zero point falls between 1.3 to 1.7 times the transverse dimension of the sample. Therefore, as the longitudinal dimension of the sample decreases, the proportion of the influence range of end effects increases, leading to a greater impact on mechanical parameters such as sample strength. Conversely, as the longitudinal dimension increases, the proportion of the end effects' influence range decreases, resulting in a gradual reduction in their impact on the sample. At the same time, reference^32^ points out that although increasing the longitudinal dimension of the sample is advantageous for suppressing end effects, the inhibitory effect weakens when the longitudinal dimension increases to 3.5 times the transverse dimension.

### The influence of displacement constraints on end effect

**Figure 18:**
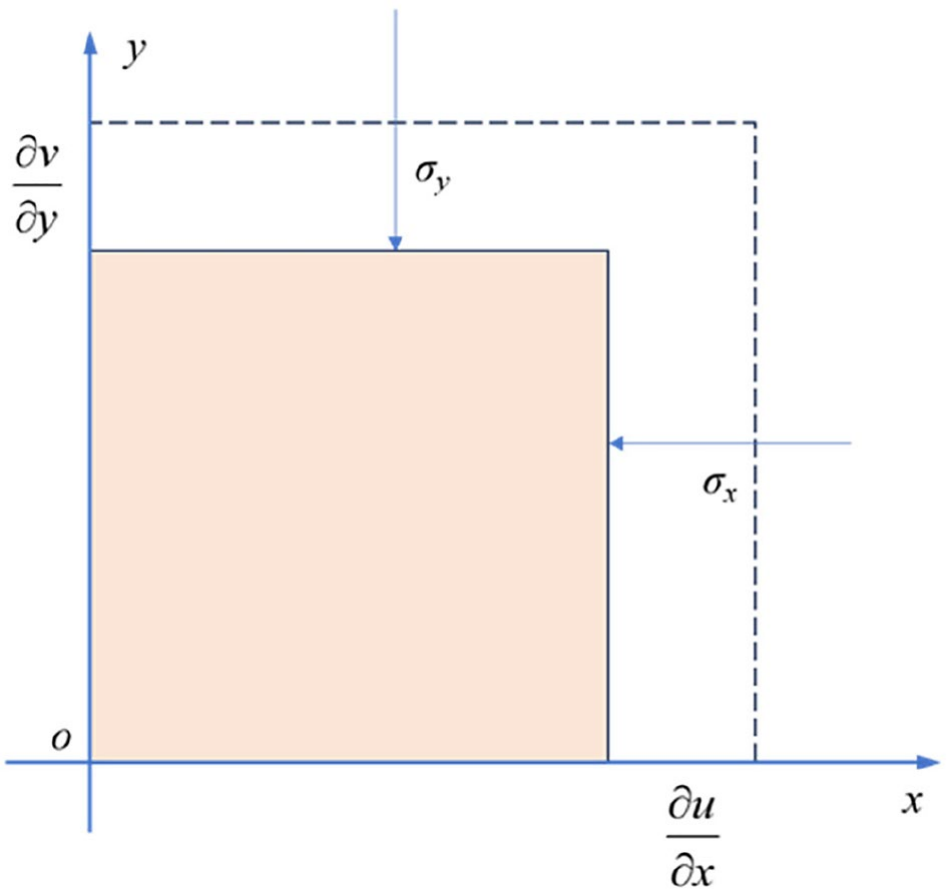
The mechanical model of the end effect influenced by lateral constraints.

To analyze the influence of end constraints on horizontal stress, a mechanical model is established as shown in Fig. 18:

16 $$ \sigma_{{\text{x}}} = \sigma_{y} = E_{r} (\frac{{u_{x,r} - u_{x,m} }}{D}) $$

In Eq. (16):

17 $$ u_{x,r} = \varepsilon_{x,r} D,u_{x,m} = \varepsilon_{x,m} D_{m} $$

18 $$ \varepsilon_{x} = - v\varepsilon_{z} = \frac{{ - v\sigma_{z} }}{E} $$

The horizontal stress induced by end constraints is given by:

19 $$ \sigma_{x} = - \frac{{E_{r} \sigma_{z} }}{D}\left( {\frac{{\nu_{r} }}{{E_{r} }} - \frac{{\nu_{m} }}{{E_{m} }}} \right) $$

In Eq. (19), *σ*_*x*_, *σ*_*y*_, and *ε*_*x*_, ε_y_ are the stresses and strains in the x and y directions, respectively. *ε*_*z*_ represents the axial strain. *ν*_r_ and *ν*_*m*_ are the Poisson's ratios of the rock sample and platen, while *E*_*r*_ and *E*_*m*_ are their respective elastic moduli. *D* represents the side length at the end of the rock sample.

As indicated by Eq. (19), under the condition of a constant elastic modulus for the sample, the transverse stress at the sample's ends is positively correlated with the platen's elastic modulus. Therefore, reducing the elastic modulus of the testing machine's platen appropriately can decrease the transverse stress at the ends of the sample.

In conclusion, as indicated by Eqs. (15) and (19), sample size and platen elastic modulus are crucial parameters influencing the end effects in the uniaxial compression process. To mitigate the impact of end effects on the uniaxial compression process, it is advisable to increase the sample height appropriately and choose platen materials with lower elastic modulus. For instance, placing rubber pads with lower elastic modulus and higher strength at the upper and lower ends of the sample can be considered to attenuate the influence of end effects on mechanical parameters.

**Figure 19:**
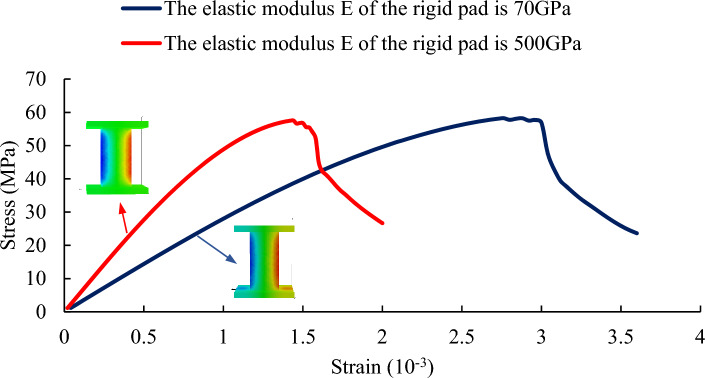
The Influence of Different Pad Stiffness on the Stress–Strain Curve of Rock Sample H100.

From Fig. 19, it can be observed that rock samples with different stiffness platen all exhibit elastic vertebral body, and the uniaxial compressive strength of the rock samples is unaffected by the stiffness of the platen. However, the stiffness of the platen has a significant impact on the elastic modulus of the rock samples. As the platen stiffness increases, the elastic modulus of the rock sample decreases. Additionally, lower platen stiffness delays the occurrence of failure in the rock sample.

### The influence of end effect on the strength of rock sample

**Figure 20:**
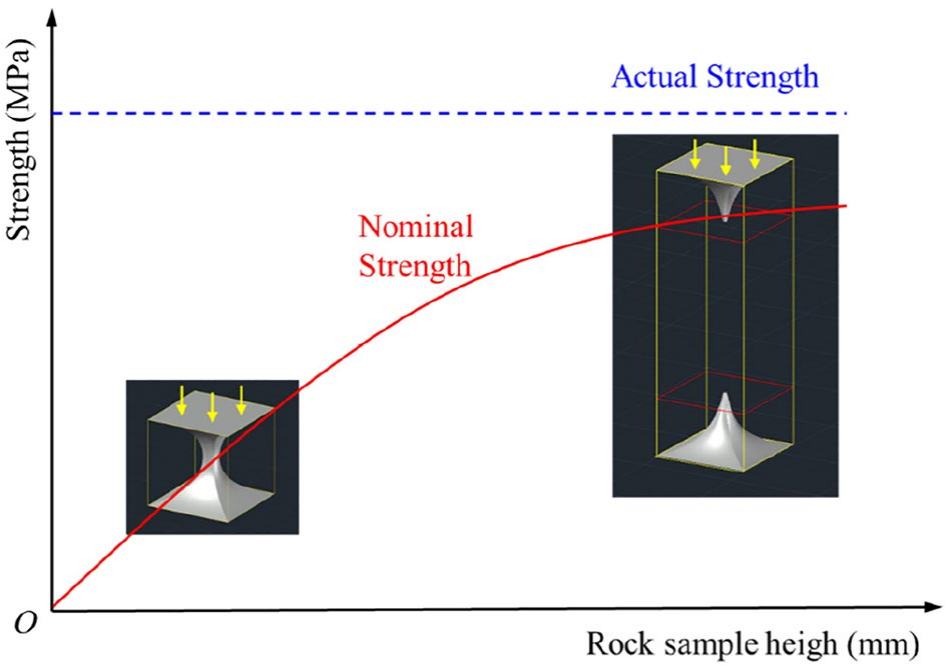
The diagram of relationship between rock sample size and strength and the distribution of elastic vertebral body.

Due to the presence of end effects during the uniaxial compression process, the strength of the rock is characterized by two concepts: actual strength (strength unaffected by changes in rock size) and nominal strength (strength of rock samples of different sizes). Numerical simulation tests reveal that, influenced by end effects, the nominal strength of the rock is less than the actual strength. However, with an increase in the longitudinal size of the rock sample, the nominal strength gradually approaches the actual strength (see Fig. 20).

Certainly, the study of size effect cannot be fully explained solely by one or a few mechanical parameters. When the probability distribution of defects within a rock sample is high, the nominal strength of the sample is influenced by the distribution of these defects, resulting in a decrease in strength as the sample size increases. For the rock samples in this study, characterized by low defect content, high density, and a large aspect ratio, the end effect on the sample become the primary factor influencing nominal strength. In the case of rock samples with a large height-to-width ratio, the strength of the sample increases with height due to the uniform uniaxial compression state in the central part, away from the influences of the ends.

During the uniaxial compression process, the end effect is influenced by the stiffness of the testing machine platen and the size of the rock sample. The disparity of stiffness between the rock sample and the platen and the roughness of the contact surface directly affect the strength of the end effect.

The end effect has a significant impact on the strength, failure mode, and energy release of small sized rock samples. However, for large sized rock samples, the central part is far from the ends, the influence of the end effect is minimal, resulting in stable variations in strength, failure modes, and energy release. The H150 rock sample exhibited a significantly higher number of AE events during failure compared to other samples, indicating a substantial increase in the number of internal unit failures. Large sized rock samples, due to the uniform compression state in the central part and minimal influence from the end effect, experience a considerable amount of shear failure, with a corresponding higher proportion of dissipative energy.

## Conclusion

- 1. With an increase in the height of rock samples, the dissipation energy density gradually rises, while the uniaxial compressive strength of the rock initially increases and then tends to stabilize. Because of the influence by the end effect, vertical cracks are observed in all rock samples upon failure.
- 2. Prior to reaching the uniaxial compressive strength, there is a noticeable fluctuation in both the quantity and energy of AE, with their peak values occurring during the post-peak stress decline phase.
- 3. When the height of rock samples is between 50 and 125 mm, the maximum values of AE quantity and energy for each type of rock sample exhibit a nearly linear increase. Upon increasing the height of the rock samples to 150 mm, a substantial increase is observed in the maximum values of AE quantity and energy, indicating an intensified level of instantaneous rock failure.
- 4. With rigid end pads at both ends of the rock sample, a noticeable elastic vertebral body is observed during loading, contrasting with its absence in the absence of rigid end pads. The load-bearing capacity of rock samples with rigid end plates exceeds that of samples without them, while the release of energy during instantaneous failure in samples without rigid end plates surpasses that in samples with rigid end plates. Before reaching the peak strength, the elastic vertebral body may exhibit interface blurring and volume collapse phenomena.
- 5. Rock samples of small size with a low height-to-width ratio are highly sensitive to the end effect, while larger samples with a greater height-to-width ratio experience weaker end effects due to the uniform compression state in the central part of the samples. To mitigate the influence of end effects on test results, rubber pads of the same size as the rock sample ends can be placed during the testing process.

## Data Availability

The data used to support the findings of this study are available from the corresponding author upon request.
